# Deaths from Chloroform

**Published:** 1877-03

**Authors:** 


### ARTICLE VI.

### *Deaths from Chloroform.*

The discussion of the relations of chloroform to dentistry is not apt to lose interest for the want of new facts bearing upon it. With the recent Rahway tragedy still fresh in our minds, we are called upon to record another victim to the administration of chloroform, while in a dentist's chair. In this instance the case was that of the wife of a prominent citizen of Rock Island, Iowa. As far as we can learn from the report furnished us, every precaution, save allowing the patient to sit in the chair, was taken to guard against the accident. The gentleman who administered the anæsthetic was an experienced physician, and had performed a similar service to the patient before. Death appeared to be instantaneous from paralysis of the heart. If it were necessary to prove the dangerous character of chloroform as an anæsthetic in dentistry, we could hardly select a more direct case in point than the one under consideration. In the Rahway case the patient had a full stomach at the time of the accident, and there were other circumstances which more or less directly invited the issue; but in the Rock Island case there would seem to have been every chance for escape, except for the fact, that the chloroform was given for tooth drawing, and the patient was at the time on the dentist's chair. In this connection we must refer to still another death from chloroform, occurring as an accompaniment of an equally trifling operation. The account has just reached us in the *British Medical Journal*, January 20, 1877, of the sudden demise of a male patient, aged thirty-seven years, at the University College Hospital, while under the anæsthetic. The operation which called for its administration was the extraction of a small piece of dead bone from the stump of an arm. The patient was lying on the bed, and the chloroform was given *secundum artem.* He became suddenly unconscious, and when the dead bone was grasped, breathing ceased at once. In this instance also all the usual methods of resuscitation failed. At the autopsy fatty degeneration of the heart was discovered. Of course the latter condition explained the cause of death, but inasmuch as the man had taken ether before without any bad symptoms, the result has a very significant bearing upon the great dangers of chloroform. If these and similar experiences serve as nothing more than new and forcible illustrations of old and acknowledged facts, a particular reference to them will not be in vain.—*Editor Med. Record.*

---

